# *Rpph1* Upregulates CDC42 Expression and Promotes Hippocampal Neuron Dendritic Spine Formation by Competing with miR-330-5p

**DOI:** 10.3389/fnmol.2017.00027

**Published:** 2017-02-07

**Authors:** Yifei Cai, Ziling Sun, Huizhen Jia, Hongxue Luo, Xiaoyang Ye, Qi Wu, Yi Xiong, Wei Zhang, Jun Wan

**Affiliations:** ^1^Shenzhen Key Laboratory for Neuronal Structural Biology, Biomedical Research Institute, Shenzhen Peking University – The Hong Kong University of Science and Technology Medical CenterShenzhen, China; ^2^Division of Life Science, The Hong Kong University of Science and TechnologyHong Kong, China

**Keywords:** Alzheimer’s disease (AD), competing endogenous RNA (ceRNAs), ribonuclease P RNA component H1 (*Rpph1*), miR-330-5p, CDC42, spine formation

## Abstract

Alzheimer’s disease (AD) is a heterogeneous neurodegenerative disease. Recent studies employing microRNA-seq and genome-wide sequencing have identified some non-coding RNAs that are influentially involved in AD pathogenesis. Non-coding RNAs can compete with other endogenous RNAs by microRNA response elements (MREs) and manipulate biological processes, such as tumorigenesis. However, only a few non-coding RNAs have been reported in the pathogenesis of AD. In this study, we constructed the first competing endogenous RNA (ceRNA) network leveraging whole transcriptome sequencing and a previously studied microRNA-seq of APPswe/PS1ΔE9 transgenic mice. The underlying mechanisms for the involvement of ceRNA in AD were validated using the Dual Luciferase Reporter Assay, detection of transcription levels by quantitative RT-PCR and translation levels by Western blotting, and morphological examination in primary cultured neurons. In the ceRNA network, four lncRNAs (C030034L19Rik, *Rpph1*, A830012C17Rik, and Gm15477) and five miRNAs (miR-182-5p, miR-330-5p, miR-326-3p, miR-132-3p, and miR-484) are enriched in nine pathways and an AD-related gene pool. Among them, Ribonuclease P RNA component H1 (*Rpph1*) is upregulated in the cortex of APPswe/PS1ΔE9 mice compared to wild type controls. *Rpph1* binds to miR326-3p/miR-330-5p and causes the release of their downstream target *Cdc42*, which leads to CDC42 upregulation. This effect was disrupted upon mutation of the MRE on *Rpph1*. Moreover, overexpression of *Rpph1* increased dendritic spine density in primary cultured hippocampal pyramidal neurons, whereas knocking down of *Rpph1* had the reverse effect. In conclusion, *Rpph1* modulates CDC42 expression level in a ceRNA-dependent manner, which may represent a compensatory mechanism in the early stage of the AD pathogenesis.

## Introduction

Alzheimer’s disease is the most common cause of dementia and is the fifth leading cause of deaths in Americans over 65 years of age (Alzheimer’s Association, 2015). Genetic heterogeneity, life style and environmental factors all contribute to the development of this disease, with the formation of amyloid β (Aβ) plague being one of the hallmarks of AD pathology (Hardy and Selkoe, 2002; Mattson, 2004; Karch et al., 2014). However, the genetic basis of AD in humans remains largely unknown. Genome-wide sequencing studies have shown that protein coding genes only occupy approximately 2% of the total genes in the human genome (Salmena et al., 2011). Numerous non-coding genes, such as LncRNAs, microRNAs (miRNAs, miRs) and piRNAs, may also orchestrate the initiation and progression of AD in humans (Okazaki et al., 2002; Guttman et al., 2009; Mercer et al., 2009; Necsulea et al., 2014; Tay et al., 2014).

Long non-coding RNA was first described in 2002 and is defined as an RNA transcript longer than 200 nucleotides with no protein product (Okazaki et al., 2002). As one of the lncRNAs, *Rpph1* is well-known as an RNA subunit of RNase P, which participates in tRNA maturation (Evans et al., 2006) and has sometimes served as a reference gene (Soler-Alfonso et al., 2014). Surprisingly, *Rpph1* was differentially expressed in gastric cancer (Xia et al., 2014) and neocortical tissues of seizure patients (Lipovich et al., 2012). Therefore, *Rpph1* is unlikely to act merely as a “house-keeping enzyme” (Jarrous and Reiner, 2007), but its functions remain elusive. MiRNAs are 19–24 nucleotides single-stranded RNA that bind to target mRNAs and either silence or degrade their targets by recruiting the RISC (Gregory et al., 2005; Esteller, 2011). Considerable evidences have shown that miRNAs are actively involved in tumorigenesis and AD pathogenesis (He and Hannon, 2004; Kefas et al., 2009; Lee et al., 2009; Jeyapalan et al., 2011; Li et al., 2013; Luo et al., 2014). The competing endogenous RNA (ceRNA) hypothesis suggests that RNAs can crosstalk by binding to miRNAs through MREs and thereby prevent miRNAs from binding to their target mRNAs (Salmena et al., 2011). The ceRNA theory has been proven true in the development of cancers and AD (Poliseno et al., 2010; Jeyapalan et al., 2011; Xia et al., 2014). For example, *BACE1-AS* prevents the binding of miR-485-5p to BACE1, augments the expression of BACE1 protein, and promotes Aβ synthesis (Faghihi et al., 2010; Riva et al., 2016). More underlying ceRNA regulations in AD still need to be uncovered.

Neuronal miR-326 was reported as a tumor suppressor gene in the brain (Kefas et al., 2009), while miR-330 was reported to suppress breast cancer and colorectal cancer development by targeting CDC42 (Jeyapalan et al., 2011; Li et al., 2013). CDC42 is a member of the Rho GTPase family that is responsible for modulating actin dynamics, stimulating spinogenesis and enlarging spine heads (Racz and Weinberg, 2008; Wegner et al., 2008; Webb et al., 2015). Moreover, CDC42 is upregulated in hippocampal neurons in AD patients compared to age-matched controls (Zhu et al., 2000).

In this study, we constructed the first AD-associated ceRNA network using data from the whole transcriptome sequencing of the cortex of APPswe/PS1ΔE9 transgenic mice and a previously reported microRNA-seq database (Luo et al., 2014). *Rpph1*, serving as an lncRNA hub in the ceRNA network targeting miR-330-5p and miR-326-3p, was found to be upregulated in APPswe/PS1ΔE9 cortexes and hippocampi. Both miR-330-5p and miR-326-3p were predicted to target CDC42, which is involved in the regulation of the actin cytoskeleton pathway. Both miR-326-3p and miR-330-5p directly bind to *Rpph1*. MiR-330-5p also induces downregulation of CDC42. Furthermore, overexpression of *Rpph1* induced upregulation of CDC42 and increased dendritic spine density, while the knocking down of *Rpph1* reduced CDC42 level and impaired dendritic spine formation. Taken together, we now show that *Rpph1* competes with endogenous miR-330-5p and subsequently upregulates CDC42 to modulate actin dynamics in primarily cultured pyramidal hippocampal neurons.

## Materials and Methods

### Tissue Samples and Genotyping

APPswe/PS1ΔE9 double transgenic mice were obtained from the Model Animal Research Center of Nanjing University (Nanjing, China) and were originated from B6.Cg-Tg (APPswe/PS1ΔE9) 85Dbo/Mmjax mice of The Jackson Laboratory). C57BL/6J mice were used as wild type (WT) controls. Mice were single-housed and bred in SPF condition IVC cages under a temperature of 23°C and a humidity of 50–60% with circadian rhythm illumination. Genotyping was performed by PCR with human *APP* and *PS1* genes, while the mouse *App* gene was used as an internal control (**Supplementary Figure S1**; **Supplementary Table S1**). Phenotyping was performed by Aβ deposition immunostaining. Two- or twelve-month-old male mice, weighing 22–25 g, were anesthetized with sodium pentobarbital and sacrificed by cervical dislocation. All procedures were approved by the Animal Use and Care Committee of Shenzhen Peking University-The Hong Kong University of Science and Technology Medical Center (SPHMC) (protocol number 2011-004). Tissues from three male C57BL/6J mice aged at 2 months were used for *Rpph1* expression profile analysis.

### RNA Extraction and qRT-PCR

Total RNA was extracted using TRIzol Reagent (Sigma) according to the manufacturer’s protocol. RNA quantity was measured using a NanoDrop 2000 (Thermo Fisher Scientific). Quantitative RT-PCR was performed using the GoScript^TM^ Reverse Transcription System (Promega) in a C1000 Thermal Cycler (Bio-Rad). Glyceraldehyde-3-phosphate dehydrogenase (*Gapdh*) or β-actin was used as internal control. Relative quantification of gene expression levels was calculated by 2^-ΔΔCt^ method. All primers are listed in **Supplementary Table S1**.

### Whole Transcriptome Sequencing and Computational Analysis

RNAs from cortical tissues of two 12-month-old male APPswe/PS1ΔE9 mice and two age-matched male WT mice (body weight 23–25 g) were used for whole transcriptome sequencing (Jiang et al., 2012) on an Illumina HiSeq2500 platform with 100 bp paired sequence at the Shanghai Biochip Corporation. In total, 3 μg of purified total RNA was isolated with a ribo-zero kit, followed by strand-specific RNA seq. Briefly, first-strand cDNA synthesis was carried out using SuperScript II kit (Invitrogen, Carlsbad, CA, USA) in the presence of a hexamer random primer. Second-strand cDNA was synthesized before end-repair and dA-tailing, and DNA fragments were ligated with a TruSeq adapter and amplified with TruSeq PCR primers for sequencing. Reads that were longer than 35 nucleotides and had no more than 2 N (ambiguous nucleotides) were retained. Moreover, paired reads that mapped to the SILVA database^1^ were discarded. The cleaned reads of each sample were aligned to the mouse RNA Ensembl database^2^ by FANSe2, allowing 7 nucleotide mismatches. All unigene clusters with at least 10 mapped reads were considered as reliable transcripts. To analyze differentially expressed unigenes, the expression of each unigene of different samples was converted to CPM (count per million) by the edgeR package. Transcripts with a false discovery rate (FDR) value lower than 0.05 and a fold change over ±1.5 were categorized as differentially expressed. The data are accessible at the NCBI GEO database, accession GSE87550.

### ceRNA Network Construction

Construction of the ceRNA network included four steps: (a) Differentially expressed lncRNAs in whole transcriptome sequences were screened using a cutoff fold change ≥± 1.5 with an FDR < 0.05. To get better robustness and reliability of the network, we strictly screened differentially expressed mRNA and ncRNA with the following criteria: expression level of Gene X was recorded for each sample: A1, A2 (APP/PS1), B1, B2 (WT). If Average (A1, A2)/Average (B1, B2) > 1.50 and if Min (A1, A2)/Max (B1, B2) < 1, then eliminate Gene X; if Average (A1, A2) /Average (B1, B2) < 0.67 and if Max (A1, A2)/Min (B1, B2) > 1, then eliminate Gene X; (b) lncRNA – miRNA interactions were predicted by the DIANA lncBase (Paraskevopoulou et al., 2013a)^3^ and the following website has the latest version: http://carolina.imis.athena-innovation.gr/diana_tools/web/index.php?r=lncbasev2\%2Findex-predicted; (c) predicted miRNA targets with a Tpm < 10 in our previous miRNAseq (Data accessible at NCBI GEO database, accession: GSE55589) (Luo et al., 2014) were removed; (d) miRNA–mRNA interactions were predicted by the DIANA web server, v5.0 (Paraskevopoulou et al., 2013b), with the support of TargetScan and Miranda^4^, and TarBase v7.0 (Vlachos et al., 2015) provided miRNA–mRNA interactions with experimental support; (e) Predicted target mRNAs were classified by KEGG analysis with DAVID Bioinformatics Resources 6.7^5^ and screened in the Alzheimer’s gene pool in GeneCards^6^.

### Cell Culture and Stressor Tests

Cortical and hippocampal neurons were isolated from E16–E18 mouse brains and seeded on poly-L-lysine (Sigma)-coated Petri dishes and cultured in Neurobasal medium (Life Technologies) containing 2% B27 (Life Technologies) at 37°C in an incubator with 5% CO_2_. For dendritic spine study, hippocampal neurons were seeded in general medium with 10% FBS for the first 4 h, followed by general medium replacement for further culturing. Culture media were half-replaced every 3 days. Neuro-2a (ATCC) and HEK 293T (ATCC) cell lines were raised in DMEM (Life Technologies) plus 10% FBS (HyClone) and antibiotics at 37°C in an incubator with 5% CO_2_. To induce differentiation, Neuro-2a cells were grown in DMEM containing 0.5% FBS, 10 μM retinoic acid (RA) (Sigma) and antibiotics for more than 24 h.

Stressor tests were performed with differentiated Neuro-2a cells in differentiation medium (without RA) under the following conditions, respectively: H_2_O_2_ (Guangzhou chemical reagent factory, Guangzhou, China) 50 μM; glucose (Sigma) 25 mM; amyloid beta 1–42 1 and 4 μM (Sigma); hyperthermia in a 42°C incubator with 5% CO_2_; three control sets including general medium only, 10 μl PBS and 1 mM ammonium hydroxide. After exposure to the indicated stressors for 12 h, cells were harvested for RNA isolation. Aβ 1–42 was dissolved in 100 μl PBS with 0.2% ammonium hydroxide, and oligomers were prepared in a 37°C water bath for 12 h.

### Plasmid, Reagents, and Antibodies

Wild type and mutant *Rpph1* sequences were cloned into pcDNA4a (Invitrogen) and psiCHECK2 (Promega) vectors. MREs of miR-326-3p (5′-CCCAGAG-3′) and miR-330-3p (5′-CCCAGAGA-3′) on *Rpph1* were mutated into 5′-GCACAGAC-3′. All plasmids were prepared in endotoxin-free conditions by QIAGEN Plasmid Midi Kit (Qiagen) or Tiangen Plasmid Mini Kit (Tiangen Biotech., Beijing, China). H_2_O_2_ was from the Guangzhou chemical reagent factory, Guangzhou, China. Glucose and Aβ 1–42 were purchased from Sigma Aldrich. MiR-326-3p and miR-330-3p mimics were purchased from RiboBio, Co., Ltd. (Guangzhou, China). Rabbit polyclonal antibodies against CDC42 and β-actin were from Abcam (ab187643) and Cell Signaling Technology (4970), respectively. The secondary goat anti-rabbit IgG antibody (A9169) was from Sigma.

### Dual Luciferase Reporter Assay

Eighty percentage confluent HEK 293T cells were transfected with Lipofectamine 2000 reagent (Life Technologies). Luciferase and Renilla activity were measured by a Dual Luciferase Reporter Assay System (Promega) at 36 h after transfection. Six independent experiments were performed.

### Transfection and RNA Interference

For the transfection of miR-326-3p, miR-330-3p, *pcDNA-Rpph1-wt* and *pcDNA-Rpph1-mutant* into Neuro-2a cells, 4 × 10^5^ cells were seeded in a 35-mm dish. A 100 nM mimic or negative control was transfected using the Lipofectamine 3000 reagent (Life technologies) the next day in DMEM with 10% FBS. After 12 h, the same transfection procedure was performed again. Cells were lysed for protein collection 72 h after the second transfection. For dendritic spine study, 1.5 × 10^5^ dissected hippocampal neurons were seeded into one well of a 24-well plate. Calcium transfection was performed at DIV9 using a CalPhos^TM^ Mammalian Transfection Kit (Clontech). 1 μg *pcDNA-Rpph1* and *pcDNA* vector backbone or 100 nM *Rpph1* siRNA was co-transfected with pEGFP at a molar ratio of 4:1. Cells were fixed at DIV15. For the RNA interference study, 100 nM *Rpph1* siRNA (5′-AAGAGUGACACGCACUCAGCACGUG-3′) was transfected into Neuro-2a cells using Lipofectamine 3000 reagent, with high-GC siRNA (Invitrogen) as the negative control. SiRNA transfection efficiency was tested by transfecting Alexa fluor 555-labeled scrambled siRNA (Invitrogen) into Neuro-2a cells (**Supplementary Figure S2A**). Three siRNA candidates were employed under the same transfection protocol (**Supplementary Figure S2B**).

### Western Blotting

Protein samples were lysed in RIPA buffer with a protease inhibitor cocktail (Sangon) and 1 mM phenylmethanesulfonyl fluoride (Sigma). Proteins were quantified by Bradford protein assay and separated with 10% SDS-PAGE. Gel separated proteins were transferred to a PVDF membrane followed by Western blotting with a primary antibody at 4°C overnight and horseradish peroxidase-conjugated secondary antibody for 2 h. Signals were developed with a Western Lightning PLUS kit (NEL105001EA, PerkinElmer). Optical density was quantified by Quantity One (Bio-Rad).

### Fluorescence Immunostaining and Image Acquisition

Neurons were fixed in a 4% PFA-PBS solution for 20 min at room temperature and permeablized and blocked in PBS containing 1% bovine serum albumin, 4% goat serum and 0.4% Triton X-100 for 30 min at room temperature. Cells were stained with primary antibodies at 4°C overnight followed by fluorescence–conjugated secondary antibodies for 2 h at room temperature. Finally, cells were stained with DAPI, washed with PBS followed by deionized water and mounted with antifade mounting medium (Beyotime). Images of cortical neurons were captured by a Zeiss LSM 710 confocal microscope with a 40x objective, while those of hippocampal neurons were captured with a Z-stack, followed by maximum intensity projection with a 40x objective. Images were analyzed with Zen 2012 (Zeiss) and ImageJ (NIH).

### Statistical Analysis

Data are presented as mean ± SEM and as differences among groups. **Figures 3F**,**4B,C,E,F**, and **5B** were analyzed by ANOVA with Bonferroni analysis, while the rest of the data were analyzed by a two-tailed Student’s *t*-test. All statistical analyses were performed by the SPSS Statistics v20.0 and 24 software package (IBM). *P*-values < 0.05 were considered statistically significant.

## Results

### Identification of Seed lncRNAs in 12-Month-Old APPswe/PS1ΔE9 Transgenic Mice

**FIGURE 1:**
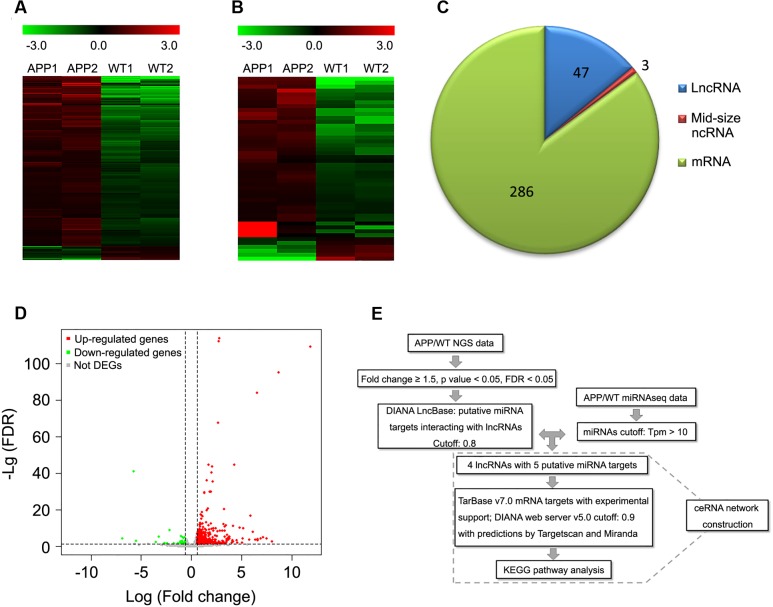
**Differentially expressed lncRNAs and mRNAs in whole transcriptome sequencing of APPswe/PS1ΔE9 transgenic mouse cortical samples**.Heat maps derived from differentially expressed mRNAs **(A)** or lncRNAs **(B)** in cortical samples of 12-month-old APPswe/PS1ΔE9 mice (APP) and wild-type mice (WT) under unsupervised hierarchical clustering. Upregulated genes are represented in red, while down-regulated genes are represented in green. **(C)** Pie chart of differentially expressed lncRNAs, mid-size mRNAs and mRNAs. **(D)** Volcano plot representing up-regulated (red), down-regulated (green) and not differentially expressed genes (DEGs) (gray). **(A–D)** Were screened under the following cutoffs: fold change ≥ ± 1.5 with FDR < 0.05. **(E)** Flowchart of ceRNA network construction. (i) Differentially expressed lncRNAs were screened by cutoffs of fold change ≥ ± 1.5 with FDR < 0.05. (ii) lncRNA–miRNA interaction was predicted by DIANA LncBase. (iii) miRNAs were removed when Tpm was less than 10 in APPswe/PS1ΔE9 miRNAseq. (iv) Potential mRNA targets of miRNAs were predicted by TarBase v7.0 and the DIANA web server v5.0 with support of TargetScan and Miranda. (v) KEGG analysis was performed among the mRNA targets.

To identify the potential contribution of lncRNAs in AD, we conducted whole transcriptome sequencing in cortical samples of 12-month-old APPswe/PS1ΔE9 transgenic mice. Overall, 47 lncRNAs, 3 mid-size ncRNAs and 286 mRNAs were found to be differentially expressed in APPswe/PS1ΔE9 cortical samples compared to WT controls (fold change ≥± 1.5, FDR < 0.05) (**Figures 1A–D**; **Supplementary Tables S2** and **S3**).

### LncRNA–miRNA Interaction Prediction

Given the hypothesis that RNAs may crosstalk through MREs as an RNA language, we next predicted lncRNA–miRNA interactions using the DIANA LncBase. Specifically, 47 differentially expressed lncRNA Ensembl gene IDs were inputted into the DIANA LncBase, and four of them obtained nine predicting miRNA targets (filter cutoff 0.8). To interrogate the roles of these predicted target miRNAs in AD pathogenesis, we utilized a previously published miRNAseq data set of APPswe/PS1ΔE9 transgenic mice (Luo et al., 2014) and those with transcripts per million (Tpm) less than 10 were removed. As a result, we obtained four seed lncRNAs targeting five miRNA targets (**Figure 1E**; **Supplementary Table S4**).

### Identification of Potential mRNA Targets of miRNAs Enriched in Signaling Pathways and an AD-Related Gene Pool

**FIGURE 2:**
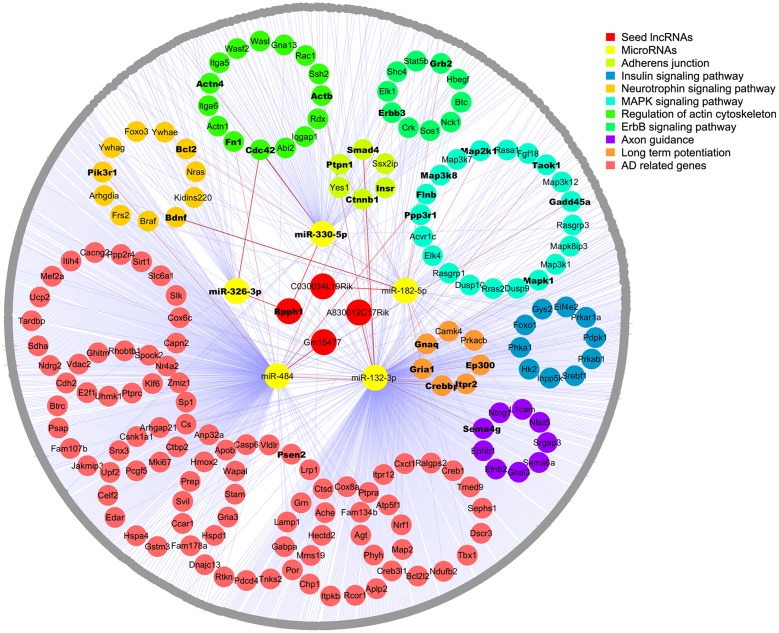
**Competing endogenous RNA network in the APPswe/PS1ΔE9 transgenic mouse model**.Four lncRNAs (red) and five miRNAs (yellow) are shown in the center; mRNAs enriched in the top eight KEGG pathways are colored and circled. The rest of the mRNA targets are shown in gray. Edges representing interactions. AD-related genes enriched in KEGG pathways are shown in bold inside dots and the gene names of gray dots are listed in the **Supplementary Material**.

To further establish the lncRNA-miRNA-mRNA interaction network, we first searched valid mRNA targets of miRNAs by TarBase v7.0 with experimental support. We then predicted potential mRNA targets through the DIANA web server v5.0 with the support of Miranda and TargetScan (filter cutoff 0.9). In total, 1082 mRNA targets were inputted into DAVID for KEGG pathway analysis, and 173 of them were enriched in the top eight AD-related pathways as following: adherent junction, insulin signaling pathway, focal adhesion, neurotrophin signaling pathway, MAPK signaling pathway, regulation of actin cytoskeleton (FDR < 0.05, *p* < 0.05), ErbB signaling pathway, and long-term potentiation and axon guidance (*p* < 0.05). Next, these mRNA targets were screened in the AD-related gene pool annotated by GeneCards, and 113 genes that were not involved in KEGG pathways were listed as AD-related genes (**Figure 2**; **Supplementary Tables S4** and **S5**).

### Construction of the ceRNA Network

Competing endogenous RNA network was constructed to include 4 seed lncRNAs, 5 miRNAs, and 1082 mRNAs. Moreover, the top eight enriched AD-related signaling pathways and the AD-related gene pool were outstanding in the network, except for focal adhesion, which shared identical genes with the other pathways. Overall, 1091 nodes with 1117 edges were found. We found that some mRNAs interact with two or more miRNAs in the network and that some mRNAs were enriched in more than one signaling pathways as well as the AD-related gene pool. To simplify the network, each mRNA is only shown in one signaling pathway, and those appear in multiple pathways or in the gene pool are shown in bold (**Figure 2**). More detailed information is shown in **Supplementary Tables S4**–**S6**.

### *Rpph1* Is Upregulated in APPswe/PS1ΔE9 Mice and by Stressor Treatments

**FIGURE 3:**
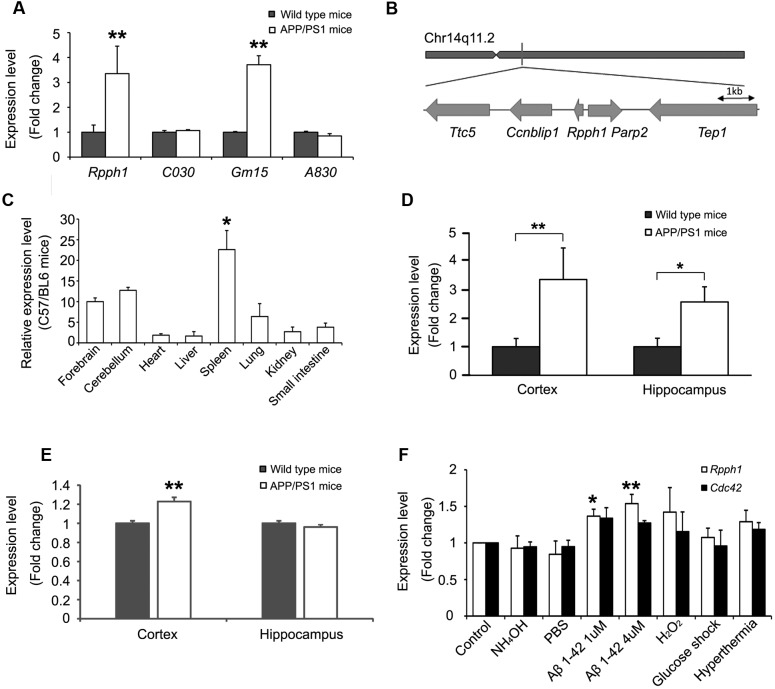
***Rpph1* was upregulated in APPswe/PS1ΔE9 mice and when under stressor treatments.(A)** Expression level of hub lncRNAs in 12-month-old APPswe/PS1ΔE9 and WT mouse cortical samples (biological replicates *n* = 17). **(B)** Genomic location of *Rpph1* and the adjacent protein coding genes. **(C)** *Rpph1* expression profile in adult C57/BL6 mouse tissues. *Rpph1* **(D)** and CDC42 **(E)** expression level in 9–12-month APPswe/PS1ΔE9 and WT mouse cortices (biological replicates *n* = 17) and hippocampal (biological replicates *n*= 14) samples. **(F)** qRT-PCR analysis of the *Rpph1* and *Cdc42* expression levels of stressor treatments in the Neuro-2a cell line. Different vehicles including culture medium (control), NH_4_OH and PBS were used as negative controls. Quantitative RT-PCR analyses were normalized to Gapdh as the internal control. ANOVA with Bonferroni analysis was performed, *F* = 7.692, ^∗∗^*p* < 0.01, ^∗^*p* < 0.05.

The expression levels of the four hub lncRNAs in 12-month-old APPswe/PS1ΔE9 and WT mouse cortical samples were examined. Expression levels of both *Rpph1* and Gm15477 (abbr: Gm15) were upregulated (**Figure 3A**). Given the hypothesis that the more MREs a RNA has the more important role it may play in the ceRNA network, we further studied *Rpph1*, which was predicted to interact with both miR-326-3p and miR-330-5p. *Rpph1* is located on Chr14q11.2 of the mouse genome (**Figure 3B**). To study the expression profile of *Rpph1*, we tested *Rpph1* RNA levels in the organs of three 2-month-old C57/BL6 mice. Our results showed that *Rpph1* is widely expressed in different organs and is abundantly expressed in the brain, lung and spleen (**Figure 3C**), consistent with previously reports (Ame et al., 2001). In our study, *Rpph1* expression level in the spleen is significantly higher than that in other organs (ANOVA with Bonferroni analysis, *F* = 6.496, 0.01 < *p* < 0.05). We examined *Rpph1* RNA levels in cortical and hippocampal samples of 9–12-month-old APPswe/PS1ΔE9 and WT mice using quantitative RT-PCR. *Rpph1* level was elevated in both cortical and hippocampal samples of APPswe/PS1ΔE9 transgenic mice compared to those of WT mice (**Figure 3D**). Of note, its predicted downstream target, *Cdc42*, was also upregulated in cortical samples in APPswe/PS1ΔE9 mice compared to those of WT (**Figure 3E**). Since multiple cell stressors have been implicated in the pathogenesis of AD (Tong et al., 2005; Borghi et al., 2007), we further asked which factors can lead to an elevation of *Rpph1* RNA levels. Differentiated Neuro-2a cells were exposed to hyperthermia, hydrogen peroxide (H_2_O_2_), high glucose, 1 or 4 μM Aβ 1–42, PBS and ammonium hydroxide for 12 h. *Rpph1* levels were upregulated to 1.37- and 1.54-fold following exposure to 1 and 4 μM Aβ 1–42, respectively (**Figure 3F**), compared to general medium treatment (control). Similar results were obtained with PBS and 1 mmol/L ammonium hydroxide treatment. These results suggest that Aβ 1–42 is the major factor reinforcing the upregulation of *Rpph1* in AD. *Cdc42* showed no significant change in the stressor test.

### *Rpph1* Modulates CDC42 Expression Level through miR-330-5p

**FIGURE 4:**
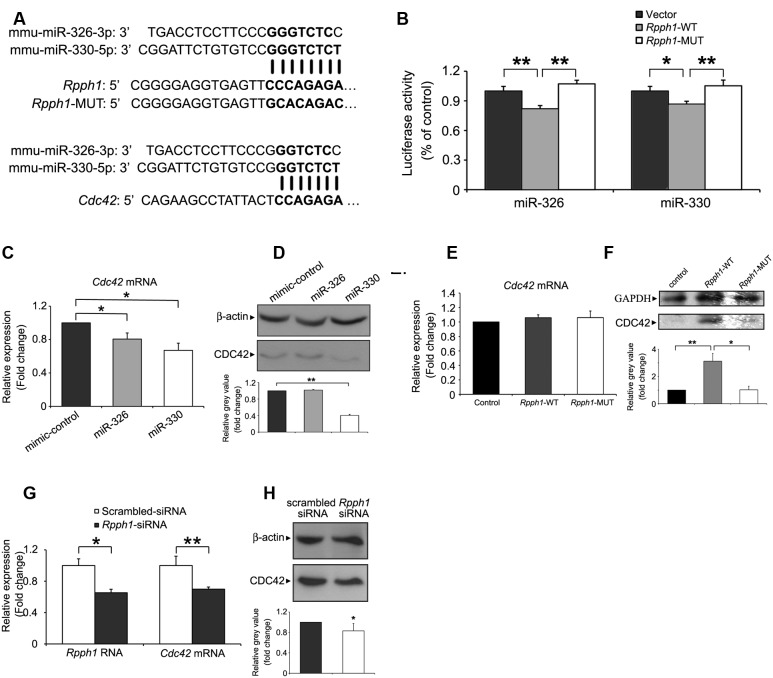
**Targeting the expression of *Rpph1* and CDC42 by miR-326-3p and miR-330-5p.(A)** Predicted binding sequences of miR-326-3p and miR-330-5p to *Rpph1* and CDC42 are shown in bold, as are the mutated sequences in the seed region of *Rpph1*. **(B)** Relative Renilla/luciferase luminescence of a psiCHECK2 vector construct harboring *Rpph1* or mutant *Rpph1* co-transfected with miR-326-3p/miR-330-5p in the HEK 293T cells, with empty psiCHECK2 vector as control. Under the condition of overexpression of miR-326-3p, p (vector/*Rpph1*-WT) = 0.009, p (*Rpph1*-WT/*Rpph1*-MUT) = 0.004, *F* = 13.048, while under the condition of overexpression of miR-330-5p, p (vector/*Rpph1*-WT) = 0.033, p (*Rpph1*-WT/*Rpph1*-MUT) = 0.005, *F* = 9.518. The data were analyzed by ANOVA with Bonferroni analysis. Relative CDC42 mRNA **(C)** or protein **(D)** levels in the Neuro-2a cell line following overexpression of miR-326-3p or miR-330-5p mimic compared to the negative control. Relative CDC42 mRNA **(E)** or protein **(F)** levels in the neuro-2a cells following overexpression of *Rpph1*. P (wt/control) = 0.006, p (wt/mutant) = 0.023, *F* = 8.066. Relative *Rpph1* and *Cdc42* mRNA levels **(G)** or CDC42 protein levels **(H)** were determined following transfection of siRNA-*Rpph1* or control siRNA in Neuro-2a cells. Quantitative RT-PCR and Western blotting in **(C–F)** were normalized to β-actin or GAPDH. Three independent experiments were performed in all tests. ^∗∗^*p* < 0.01, ^∗^*p* < 0.05.

To examine lncRNA–miRNA interactions in the ceRNA network, we predicted possible MREs in *Rpph1* and identified miR-326-3p and miR-330-5p as potential targets. MiR-330-5p shares eight nucleotide binding sequences with *Rpph1* while miR-326-3p shares seven nucleotide binding sequences (**Figure 4A**). We next cloned WT and mutant *Rpph1* into the psiCHECK2 vector. Plasmids were transfected into HEK 293T cells with empty psiCHECK2 plasmid as a control. Results from Dual Luciferase Reporter Assay showed that both miR-326-3p and miR-330-5p inhibit *Rpph1* WT luminescence activity by 20%. The reduction in luciferase activity was not seen when miRNA binding sequence was mutated (**Figure 4B**). No significant difference was observed between vector control and *Rpph1*-MUT. These results suggest that miR-326-3p and miR-330-5p bind to *Rpph1*.

As one miRNA can target various mRNAs, we searched for the mutual protein coding mRNA targets of miR-326-3p and miR-330-5p. Twelve mutual mRNAs were found, among which Cdc42 and Itga5 were implicated in the regulation of actin cytoskeleton. MiR-330-5p has been reported to target CDC42 in human breast cancer cell line MT-1 (Jeyapalan et al., 2011) and colorectal cancer cell SW1116 (Li et al., 2013). Moreover, CDC42 expression level is upregulated in hippocampal pyramidal neurons of AD patients (Zhu et al., 2000). To this end, we further pursued the interaction between *Rpph1*, miR-326-3p/miR-330-5p and CDC42. To confirm that miR-326-3p and miR-330-5p target CDC42 in the neural system, we transfected miRNA mimics and negative controls into Neuro-2a cells and examined the levels of CDC42 mRNA and protein. *Cdc42* mRNA levels were decreased by overexpression of miR-330-5p and miR-326-3p to 37.2 and 20%, respectively (**Figure 4C**). CDC42 protein level was markedly inhibited by miR-330-5p, while miR-326-3p overexpression did not show a significantly inhibitive effect (**Figure 4D**).

We then tested whether *Rpph1* could modulate *Cdc42* mRNA and protein levels. To this end, we cloned WT and mutant *Rpph1* sequence into a pcDNA4A vector and transfected these constructs into Neuro-2a cells. Overexpression of *Rpph1* resulted in significantly elevated level of CDC42 protein (**Figure 4F**) without affecting its mRNA level (**Figure 4E**). Such effect of increased CDC42 protein was not observed when cells were transfected with mutant *Rpph1* (**Figure 4F**). These results suggest that *Rpph1* promotes the expression of CDC42 by impeding miR-330-5p binding. Consistent with this notion, transfection of *Rpph1* siRNA resulted in decreased levels of CDC42 mRNA and protein (**Figures 4G,H**).

### *Rpph1* Promotes the Development of Dendritic Spine Density in Hippocampal Pyramidal Neurons through Upregulation of CDC42

**FIGURE 5:**
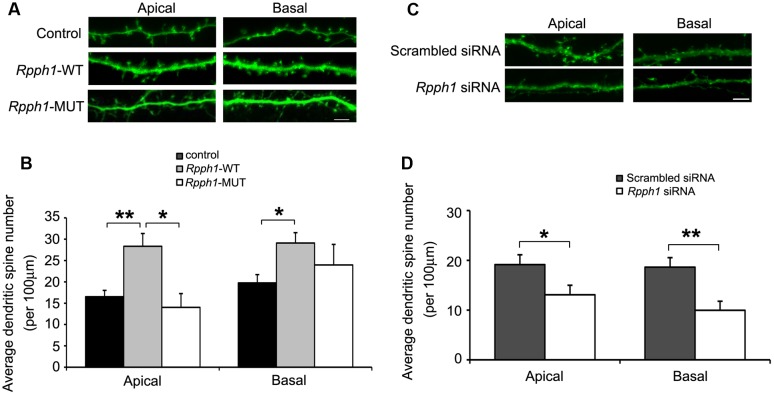
***Rpph1* enhances dendritic spine density in hippocampal pyramidal neurons**.Apical and basal dendritic spines that extended 50–150 μm of the soma were observed in pyramidal neurons **(A,C)**. The average dendritic spine number per 100 μm of dendrites was shown following overexpression of WT or mutant *Rpph1* **(B)** or knocking down of *Rpph1* **(D)** in both the apical and basal dendrites of mouse hippocampal pyramidal neurons. **(A,B)** Apical dendritic spine: p (wt/control) = 0.001, p (wt/mutant) = 0.027, *F* = 8.385; basal dendritic spine: p (wt/control) = 0.018, p (wt/mutant) = 0.45, *F* = 4.022). **(C,D)** p (apical) = 0.042, p (basal) = 0.002. Scale bar = 5 μm, 10–15 transfected neurons were randomly selected in each experiment. Three independent experiments were performed. ^∗∗^*p* < 0.01, ^∗^*p* < 0.05.

As *Rpph1* is upregulated in 9–12-month-old APPswe/PS1ΔE9 cortices and hippocampi, we hypothesized that it may influence neuronal behavior. To investigate whether the elevation of the *Rpph1* level could lead to any phenotypic changes in neuronal cells, we examined dendritic spine formation in cultured primary hippocampal pyramidal neurons. DIV9 hippocampal neurons were co-transfected with *pcDNA- Rpph1* and *pEGFP* and fixed at DIV15. Dendritic spines that extended 50–150 μm from the soma were observed by confocal microscopy and counted as two separate groups: apical dendrites and basal dendrites. Both apical and basal dendritic spine numbers were increased following the overexpression of *pcDNA- Rpph1* (apical dendritic spine: 28.34 ± 2.98 versus 16.53 ± 1.48; basal dendritic spine: 29.09 ± 2.43 versus 19.75 ± 1.95, Mean ± SEM, *n* = 18–25, ^∗∗^*p* < 0.01) (**Figures 5A,B**). This effect was reversed under overexpression of *pcDNA-Rpph1-mutant* (**Figures 5A,B**). Similar decrease in dendritic spine formation was observed when we knocked down *Rpph1* using siRNA (**Figures 5C,D**).

## Discussion

Recent studies suggest that RNAs, including lncRNAs, circRNAs, pseudogenes, and mRNAs (Fatica and Bozzoni, 2014), can function as miRNA sponges (Cesana et al., 2011; Kallen et al., 2013; Wang et al., 2013) and endogenously compete with each other through MREs to modulate disease processes (Salmena et al., 2011; Tay et al., 2014), including that of AD (Faghihi et al., 2008; Mercer et al., 2009; Esteller, 2011). Apart from post-transcriptional regulation, epigenetic modifications, such as histone acetylation, also pose a critical role in AD pathogenesis (Chouliaras et al., 2010; Rudenko and Tsai, 2014), making AD a highly complex disease to study. These refined regulatory networks may explain why the isolation of a single component (e.g., β-secretase and apolipoprotein E4) failed to fully explain the whole pathogenesis process of AD (Mullane and Williams, 2013; Spinney, 2014). In this study, we constructed the first AD-related ceRNA network based on the APPswe/PS1ΔE9 transgenic mouse model. A number of AD-related genes were also found in the ceRNA network. Taken together, our analysis suggests potential pathways in a comprehensive ceRNA network, which sheds light on the unknown regulatory pathways in AD. To obtain a better robustness and reliability of the network, we strictly restrained the cutoff values for gene entities and screened mRNA candidates using multiple databases with validated experimental supports. Nonetheless, imperfections still remain in the lncRNA–miRNA interaction prediction databases. For example, a considerable amount of lncRNA inputs did not link with miRNA outputs, which could lower the network’s sensitivity. It is noteworthy that the expression levels of both miR-326-3p and miR-330-5p did not change significantly compared to our previous microRNA-seq study (Luo et al., 2014). In a ceRNA pathway, microRNAs act as mediators between upstream and downstream RNAs, either markedly increasing or decreasing microRNA levels, which may disrupt the balance of ceRNA crosstalk.

*Rpph1* is well-known as an RNA subunit of RNase P, which is responsible for tRNA maturation in all three domains of life: from Achaea to Bacteria and Eukarya (Evans et al., 2006). *Rpph1* has also been used to as an internal control for RNA quantification (Raoul et al., 2005; Page et al., 2011; Soler-Alfonso et al., 2014) However, recent deep sequencing studies showed that *Rpph1* was up-regulated in the human gastric cancer tissues (Xia et al., 2014) and in the neocortex of seizure patients (Lipovich et al., 2012), as well as in cortical samples from the APPswe/PS1ΔE9 mice. Moreover, a biochemical study has shown that RNase P takes part in lncRNA *MALAT1* maturation (Tripathi et al., 2010). The data suggest that *Rpph1* may be involved in the processes of disease progression in animals and humans.

Cell division cycle 42 is a member of the Rho family small guanosine triphosphatases (GTPases) that are involved in cell morphology, migration, and cell cycle progression (Nobes and Hall, 1995; Qiu et al., 1997; Hall, 1998). Multiple lines of evidence show that elevated CDC42 in neurons promotes neurite outgrowth and dendritic spine formation (Brown et al., 2000; Kreis et al., 2007). Synaptic strength and neuronal functions are largely influenced by the size and number of dendritic spines (Penzes and Vanleeuwen, 2011). Notably, researchers have found that CDC42 is upregulated in the hippocampal neurons of Alzheimer’s patients compared to age-matched controls (Zhu et al., 2000).

Synaptic scaling is a compensatory homeostatic mechanism in order to maintain the excitatory response of individual neurons by preventing the catastrophic amnesia associated with synaptic loss during AD progression. This process involves alterations in both neuronal excitability and dendritic architecture (Small, 2004; Abuhassan et al., 2014). Synapse loss is frequently observed in the postmortem brain tissues of AD patients (Scheff et al., 1990; Penzes and Vanleeuwen, 2011). Interestingly, neuropathological studies also found that compensatory changes occurring in AD brains. An example is such changes is the enlargement of the remaining dendritic spines and the consequent maintenance of total synaptic contact area (Scheff and Price, 1993; Fiala et al., 2002). Our study shows that *Rpph1* enhances the expression level of CDC42 and promotes dendritic spine formation by competing for endogenously expressed miR-330-5p. This regulatory loop represents a potential compensatory mechanism in the early stage of AD pathogenesis (Penzes and Vanleeuwen, 2011; Guo et al., 2013).

## Conclusion

We constructed the first ceRNA network based on the APPswe/PS1ΔE9 transgenic mouse model. We propose that one of our tested ceRNA pathways, *Rpph1*/miR-330-5p/CDC42, may be involved in the compensatory behavior of the brain neurons to combat synaptic loss during AD pathogenesis. These findings provide further insight into the pathophysiological mechanism of AD.

## Ethics Statement

APP/PS1 double transgenic mice model and WT C57/BL6 and ICR mice were used in our study. All procedures were approved by the Animal Use and Care Committee of Shenzhen Peking University-The Hong Kong University of Science and Technology Medical Center (SPHMC) (protocol number 2011-004). Considerations and procedures were taken to lower the number of animals to use in the study and lowered the pain of the animals.

## Author Contributions

YC carried out the molecular and cellular studies, participated in the bioinformatics analysis and drafted the manuscript. ZS participated in the bioinformatics analysis and built up the ceRNA network with YC. HJ helped to repeat and confirm the molecular biological data. HL, XY, QW, and YX participated in the animal experiments, including genotyping, phenotype identification, tissue collection and RNA/protein extraction, and helped to revise the manuscript. WZ participated in the design of the study, performed the statistical analysis and helped to revise the manuscript. JW conceived of the study, participated in its design and coordination and helped to draft the manuscript. All authors read and approved the final manuscript.

## Conflict of Interest Statement

The authors declare that the research was conducted in the absence of any commercial or financial relationships that could be construed as a potential conflict of interest.

## Footnotes

1 http://www.arbsilva.de/download/arbfiles/

2 http://www.ensembl.org/index.html

3 http://diana.imis.athena-innovation.gr/DianaTools/index.php?r=lncPredicted/index

4 http://diana.imis.athena-innovation.gr/DianaTools/index.php?r=MicroT_CDS/index

5 https://david.ncifcrf.gov/

6 http://www.genecards.org/Search/Keyword?queryString=alzheimer

## Supplementary Material

FIGURE S1 **Genotyping of human *APP* and *PS1* genes in the APPswe/PS1ΔE9 mouse model**. Human APP and PS1 genes were amplified with designed primers (**Supplementary Table S6**) using mouse tail genomic DNA as template. The mouse *App* gene was used as an internal control. Since human APP and PS1 were introduced on the same plasmid for transgenic mouse construction, both APP and PS1 should show positive results in transgene-positive mice. “A” for APPswe/PS1ΔE9 mice, “W” for wild type mice.

FIGURE S2 **RNA interference mediated knocking down of *Rpph1* in Neuro-2a cell lines. (A)** Neuro-2a cells were transfected with Alexa fluor 555-labeled scrambled siRNA. Approximately 80% of all cell were transfected. **(B)** Three siRNA candidates for *Rpph1* were employed with scrambled siRNA (NC) and non-transfected cells (NA) as control. SiRNA2 showed a 36% knockdown, and siRNA3 showed a 20% knockdown. Three independent experiments were performed in all experiments. ^∗∗^*p* < 0.01.

Table S1 **Primer list**.

Table S2 **Differentially expressed lncRNAs**.

Table S3 **Differentially expressed mRNAs**.

Table S4 **Nodes interactions**.

Table S5 **AD related gene pool**.

Table S6 **KEGG pathways**.

## Abbreviations

AD: Alzheimer’s disease
Aβ: beta amyloid peptide
CDC42: cell division cycle 42
LncRNA: long non-coding RNAs
MiR: MicroRNA
MREs: microRNA response elements
Rpph1: ribonuclease P RNA component H1
RISC: RNAinduced silencing complex
