# Supplementary figures and images for: Rpph1 Upregulates CDC42 Expression and Promotes Hippocampal Neuron Dendritic Spine Formation by Competing with miR-330-5p
Source: Front Mol Neurosci. 2017 Feb 7;10:27. doi: 10.3389/fnmol.2017.00027 (PMC5293807; doi:10.3389/fnmol.2017.00027)

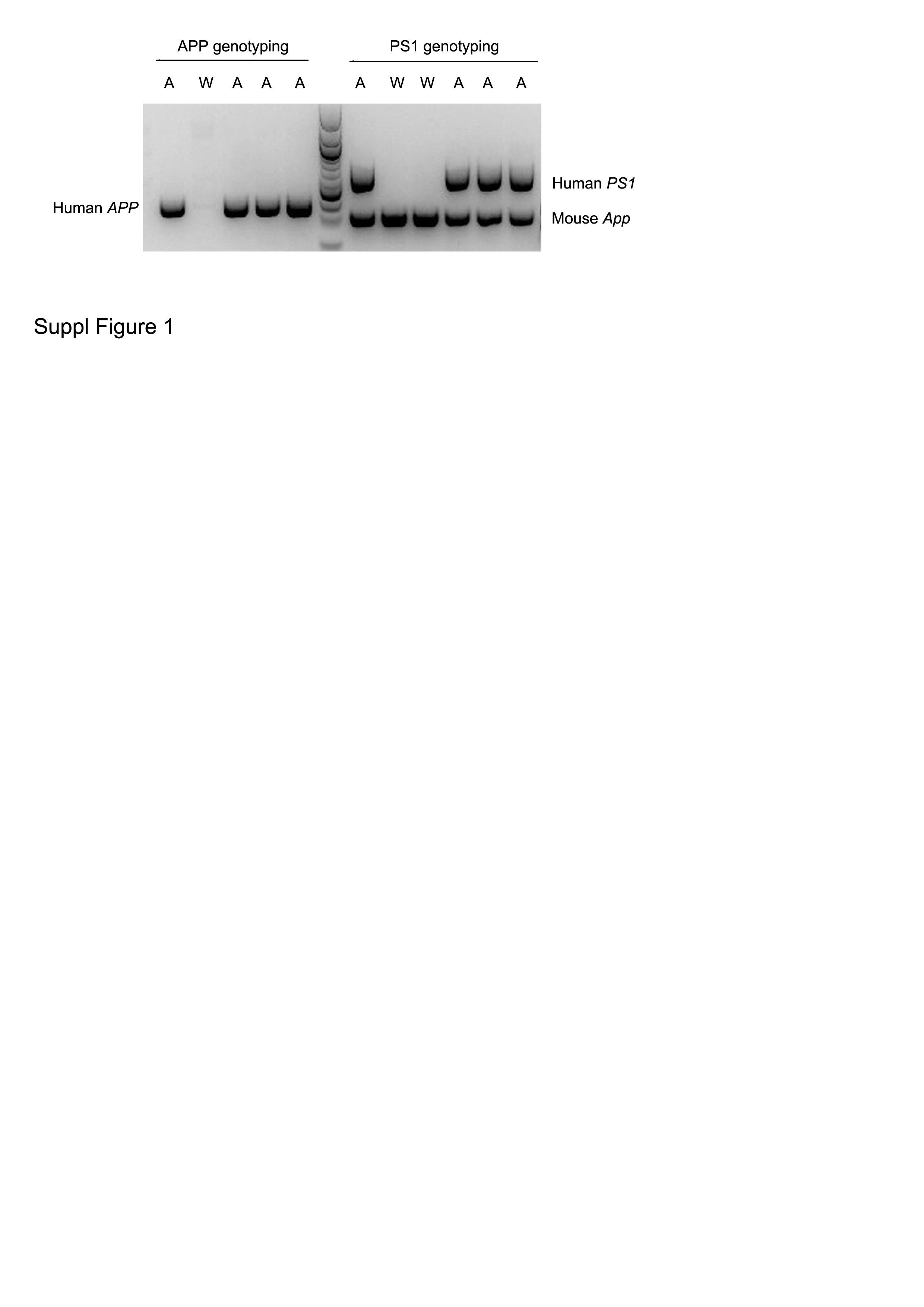

Supplement: FIGURE S1 — Genotyping of human APP and PS1 genes in the APPswe/PS1ΔE9 mouse model. Human APP and PS1 genes were amplified with designed primers (Supplementary Table S6) using mouse tail genomic DNA as template. The mouse App gene was used as an internal control. Since human APP and PS1 were introduced on the same plasmid for transgenic mouse construction, both APP and PS1 should show positive results in transgene-positive mice. “A” for APPswe/PS1ΔE9 mice, “W” for wild type mice. [file Image_1.TIF]

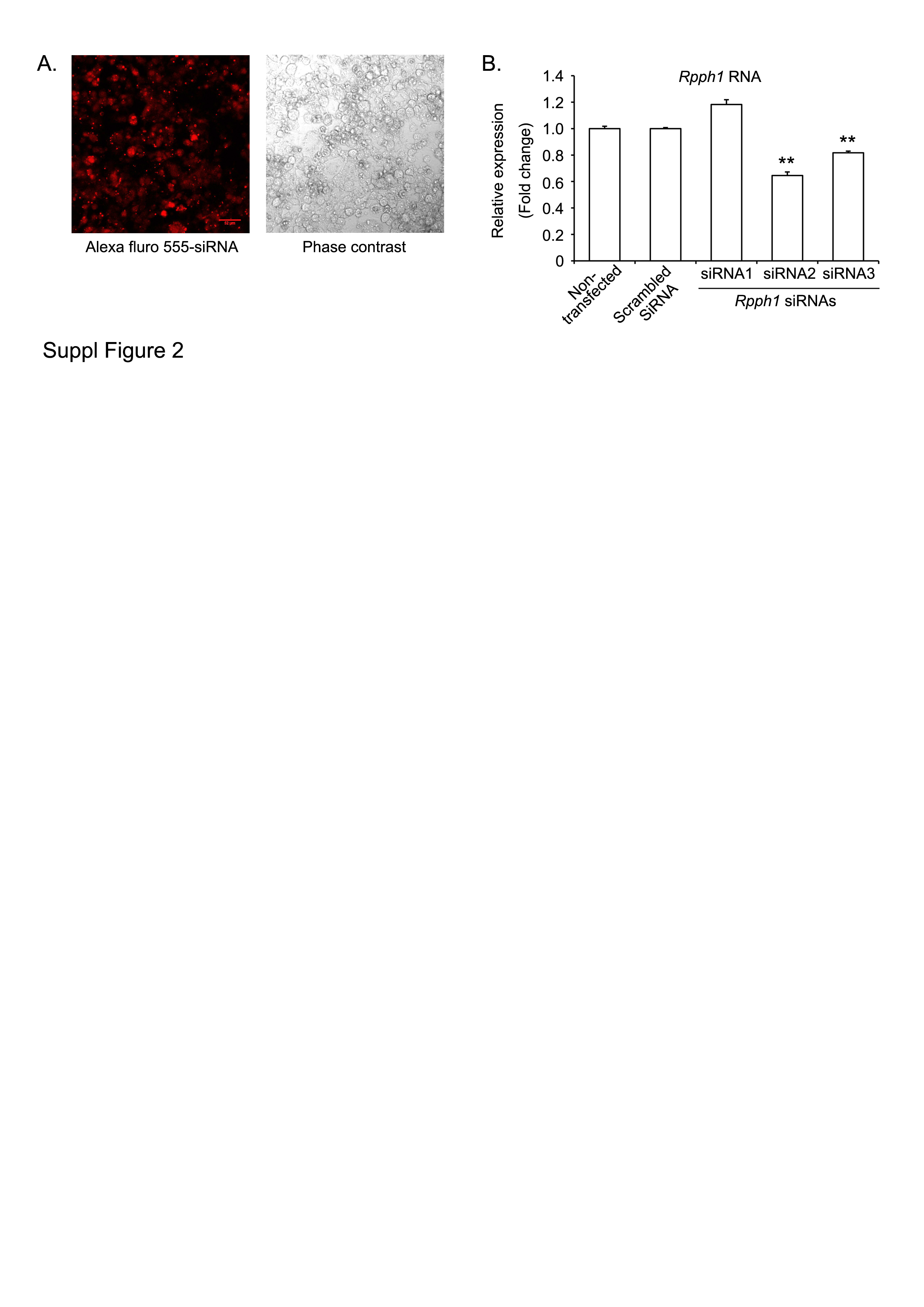

Supplement: FIGURE S2 — RNA interference mediated knocking down of Rpph1 in Neuro-2a cell lines. (A) Neuro-2a cells were transfected with Alexa fluor 555-labeled scrambled siRNA. Approximately 80% of all cell were transfected. (B) Three siRNA candidates for Rpph1 were employed with scrambled siRNA (NC) and non-transfected cells (NA) as control. SiRNA2 showed a 36% knockdown, and siRNA3 showed a 20% knockdown. Three independent experiments were performed in all experiments. ∗∗p < 0.01. [file Image_2.TIF]
