# Supplementary material for: Rpph1 Upregulates CDC42 Expression and Promotes Hippocampal Neuron Dendritic Spine Formation by Competing with miR-330-5p
Source: Front Mol Neurosci. 2017 Feb 7;10:27. doi: 10.3389/fnmol.2017.00027 (PMC5293807; doi:10.3389/fnmol.2017.00027)
Supplement: Table S1 — Primer list. [file Table_1.DOCX]

| Primer name | Primer sequence |
| --- | --- |
| *Rpph1* mouse forward | 5’ -AGTGCGTGTCACTCTTCG- 3’ |
| *Rpph1* mouse reverse | 5’ -TGCTCTCTGGGAACTCAC- 3’ |
| *RPPH1* human forward | 5’ -GAGCTGAGTGCGTCCTGTC-3’ |
| *RPPH1* human reverse | 5’ - TCAGGGAGAGCCCTGTTAGG- 3’ |
| *Gapdh* mouse forward | 5’- AACTTTGGCATTGTGGAAGG-3’ |
| *Gapdh* mouse reverse | 5’- GGATGCAGGGATGATGTTCT- 3 |
| *GAPDH* human forward | 5’- CAGCCTCAAGATCATCAGCA- 3’ |
| *GAPDH* human reverse | 5’- TGTGGTCATGAGTCCTTCCA- 3’ |
| *Cdc42* mouse forward | 5’- CCCTCACACAGAAAGGCCTAAA- 3’ |
| *Cdc42* mouse reverse | 5’- ATGCGTTCATAGCAGCACAC- 3’ |
| Beta actin mouse forward | 5’- GATCAAGATCATTGCTCCTCCTG -3’ |
| Beta actin mouse reverse | 5’- AGGGTGTAAAACGCAGCTCA -3’ |
| siRNA-*Rpph1* | 5’- AAGAGUGACACGCACUCAGCACGUG -3’ |
| Human *APP* forward | 5’- AGG ACT GAC CAC TCG ACC AG - 3’ |
| Human *APP* reverse | 5’- CGG GGG TCT AGT TCT GCA T - 3’ |
| Human *PS1* forward | 5’- AAT AGA GAA CGG CAG GAG CA - 3’ |
| Human *PS1* reverse | 5’- GCC ATG AGG GCA CTA ATC AT - 3’ |
| Mouse *APP* forward | 5’- CTAGGCCACAGAATTGAAAGATCT - 3’ |
| Mouse *APP* reverse | 5’- GTAGGTGGAAATTCTAGCATCATCC -3’ |

Primer list:
